# An Analysis of Great Tit Egg Traits Across the City Mosaic: Urbanisation Does Not Affect Egg Size and Pigmentation Patterns

**DOI:** 10.1002/ece3.73867

**Published:** 2026-07-30

**Authors:** Ignacy Stadnicki, Michela Corsini, Klaudia Szala, Andrew Gosler, Marta Szulkin

**Affiliations:** ^1^ Institute of Evolutionary Biology Faculty of Biology, Biological and Chemical Research Centre University of Warsaw Warsaw Poland; ^2^ Institute of Human Sciences, School of Anthropology & Museum of Ethnography University of Oxford Oxford UK; ^3^ Institute for Alpine Environment Eurac Research Bolzano Italy; ^4^ Department of Avian Biology and Ecology, Faculty of Biology Adam Mickiewicz University Poznań Poland; ^5^ Department of Biology George Mason University Fairfax Virginia USA; ^6^ Edward Grey Institute of Field Ornithology, Department of Biology University of Oxford Oxford UK

**Keywords:** calcium, egg pigmentation, egg volume, environmental constraints, great tit, protoporphyrin IX, reproduction, urbanisation

## Abstract

Rapid urbanisation provides remarkable opportunities to study how sudden, extreme changes impact wildlife. Compared to natural areas, cities are characterised by factors affecting abiotic (e.g., climate, habitat fragmentation) and biotic (e.g., species composition, phenology) components of the ecosystem, ultimately changing the ecological and evolutionary dynamics of those habitats. Similarly to many other taxonomic groups, urban birds differ from rural birds in morphology, behaviour and reproductive patterns. Yet potential associations between urbanisation and avian egg traits—a key aspect of birds life‐cycle—remain under‐researched. Given the limited availability of primary natural calcium sources (snails) in cities, eggs from heavily urbanised areas were expected to be smaller and more pigmented, indicating thinner shells and lower overall egg quality. To better understand how urbanisation affects egg traits, data on 718 great tit ( *Parus major* ) eggs from 90 clutches, spread across eight study sites in a city mosaic, were collected for two breeding seasons. All clutches were photographed and analysed using digital imaging and visual scoring to assess egg volume and pigmentation patterns. Urbanisation was quantified as the percentage of Impervious Surface Area (ISA) in the vicinity of each clutch via satellite imagery. In line with some studies conducted on semi‐natural bird communities, egg volume covaried with lay date and female body condition, while for both egg volume and egg pigmentation (spots percentage) a year effect was detected. However, in contrast with the predictions, there was no association between urbanisation and the examined egg traits. While urban clutches are consistently smaller, this study shows that eggs as such are similar to those found in rural habitats in terms of volume and pigmentation patterns. Thus, urban‐driven environmental pressures may not be as strong or directional during the egg laying phase as they are at later stages of reproduction.

## Graphical abstract

Based on data from over 550 great tit ( *Parus major* ) eggs collected in the city mosaic, this research finds no effect of urbanisation on egg volume or pigmentation patterns. These results suggest that urban‐driven environmental pressures may not be as strong or directional during the egg laying phase as they are at later stages of reproduction.

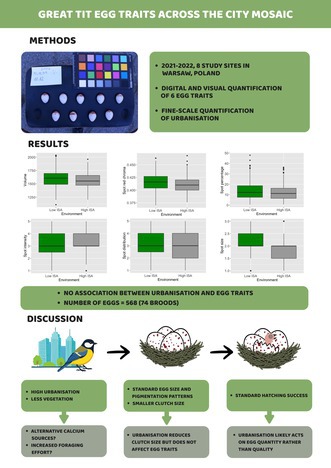

## 1 Introduction

Cities provide remarkable opportunities to study how extreme environmental changes can impact organismal biology. Impervious surfaces are a key attribute of urban areas (Szulkin et al. 2020), creating highly fragmented habitats for urban wildlife, higher average temperatures (known as the urban heat island effect; Oke 1973), lower capacity for water retention, and fewer biologically active areas. Ultimately, such changes are known to affect eco‐evolutionary dynamics, species composition, abundance, interactions (Des Roches et al. 2021), and even genetic makeup (Salmón et al. 2021).

Due to its common presence in both rural habitats and cities (Bańbura and Bańbura 2012), the great tit ( *Parus major* ) is a valuable model organism for urban biology research. Over the years, much work has demonstrated the impact of urbanisation on a large number of this passerine's traits, including changes in morphology (Thompson et al. 2022), behaviour (Corsini et al. 2022), survival (Corsini et al. 2020; Corsini and Szulkin 2025) and reproduction (Charmantier et al. 2017). For instance, urban tits start laying their clutches earlier, often produce fewer eggs (Charmantier et al. 2017), hatchlings and fledglings (Corsini and Szulkin 2025) relative to their rural conspecifics. However, only a few studies to date investigated the potential associations between urbanisation and egg size (Hõrak et al. 1995; Bańbura et al. 2010; Bailly et al. 2016; Hargitai, Nagy, Nyiri, et al. 2016) or pigmentation (Hargitai, Nagy, Nyiri, et al. 2016). More generally, great tit egg traits have been shown to be associated with the immediate environment—specifically, calcium availability (Mänd et al. 2000; Gosler et al. 2005; Gosler and Wilkin 2017) and were proposed as predictors of clutch quality (Sanz and García‐Navas 2009). Namely, egg size has been shown to positively covary with calcium availability (Mänd et al. 2000), hatching success, nestling development and survival (meta‐analysis, Krist 2011), while pigmentation patterns were found to correlate with calcium availability, eggshell thickness (Gosler et al. 2005), incubation period duration, hatching success, and nestling morphology (Sanz and García‐Navas 2009). Additionally, both traits are likely to have genetic basis (see Ojanen et al. 1979; Santure et al. 2013 for egg size and Gosler et al. 2000 for pigmentation patterns).

Great tits lay one egg per day, with clutches typically characterised by 5 to 12 eggs. Each egg weighs *c*.1.2–2.0 g (Gosler et al. 2005), and has a volume of 1.50 ± 0.12 cm^3^ (Encabo et al. 2001). The eggshell is white with reddish spots (the pigment is protoporphyrin IX, produced by females in the biosynthesis of blood haem—Burley and Vadehra 1989). The primary component of the eggshell is calcium carbonate (Romanoff and Romanoff 1949), and a clutch of a blue tit ( *Cyanistes caeruleus* ) can contain more calcium than the female's entire skeleton (Graveland 1995). To cater for this need, great tits can rapidly increase their calcium intake in preparation for egg laying, with daily consumption of snail shells starting at 4 mg in the pre‐laying period, increasing to 16 mg 2 days before clutch initiation, and peaking to an average of 65 mg throughout egg laying (Graveland and Berends 1997). Moreover, studies on house sparrows ( *Passer domesticus* ; Krementz and Ankney 1995) and zebra finches ( *Taeniopygia guttata* ; Reynolds 1997) found little to no cortical bone contribution of dietary calcium to eggshell formation, with medullary bone (a temporary and labile form of bone found in the long bones of laying females) being utilised as a short‐term store of the calcium that is depleted and replenished on a daily basis. Consequently, breeding tits primarily rely on environmental calcium intake, making it a limiting resource in egg formation (Graveland and Gijzen 1994; Graveland and Berends 1997; Graveland and Drent 1997; Tilgar et al. 2002). Hence, breeding in calcium‐poor habitats can have detrimental consequences for bird reproduction (Reynolds and Perrins 2010). The main source of calcium for great tits in rural environments (such as woodlands and forests) are small snails (Graveland 1996), which may be negatively affected by urbanisation both in terms of species diversity (Čiliak et al. 2024) and abundance (Perez et al. 2021), although abundance patterns are species‐specific (Saeki et al. 2020).

Snail abundance correlates strongly with soil‐calcium (Jubb et al. 2006). In tits, calcium availability in the immediate environment has been shown to be reflected in both egg size and pigmentation patterns in non‐urban habitat. Thus, eggs in calcium‐poor areas are smaller (Mänd et al. 2000; Hargitai et al. 2013; Bańbura et al. 2020), with pigment being darker and more aggregated around the broad end of the egg (Gosler et al. 2005; Gosler and Wilkin 2017; Briggs and Mainwaring 2017; see Figure 2 for an example of pigment variation within and between clutches).

Previous researchers did not find urban‐driven differences in great tit egg size (Hõrak et al. 1995; Bańbura et al. 2010; Bailly et al. 2016; Hargitai, Nagy, Nyiri, et al. 2016) or pigmentation patterns (Hargitai, Nagy, Nyiri, et al. 2016). However, in earlier studies urbanisation was defined using habitat dichotomy (urban vs. rural), an approach that does not necessarily reflect the ecological structure of the urban mosaic (Szulkin et al. 2020). Most importantly, to date, only one study examined pigmentation patterns in an urban–rural comparison, and was based on a small sample size (Hargitai, Nagy, Nyiri, et al. 2016; a total of 37 eggs from 37 clutches)—thus, finer scale environmental data and larger sample sizes are needed to uncover potential urban‐driven variation in egg traits.

The aim of this study is to quantify the impact of urbanisation on egg size and pigmentation patterns (proposed indicators of clutch quality), which is currently overlooked in the field of urban ecology. To achieve this goal, data were collected over 2 years on 718 eggs from 90 clutches, spread across eight diverse study sites within the urban mosaic of Warsaw, Poland and its surrounding areas. Importantly, urbanisation was quantified at high resolution with the use of remote sensing imagery, thus acknowledging the fine‐scale environmental variability occurring in cities. Considering the amount of impervious surface area (ISA) in the city—which lacks vegetation and thus cannot support snails—and the well documented detrimental impact of urbanisation on nestling development (e.g., Chamberlain et al. 2009; Bailly et al. 2016; Corsini et al. 2020), we predicted that eggs found in highly urbanised areas would be smaller and more pigmented, indicating lower clutch and habitat quality.

## 2 Methods

### 2.1 Study Sites

**FIGURE 1:**
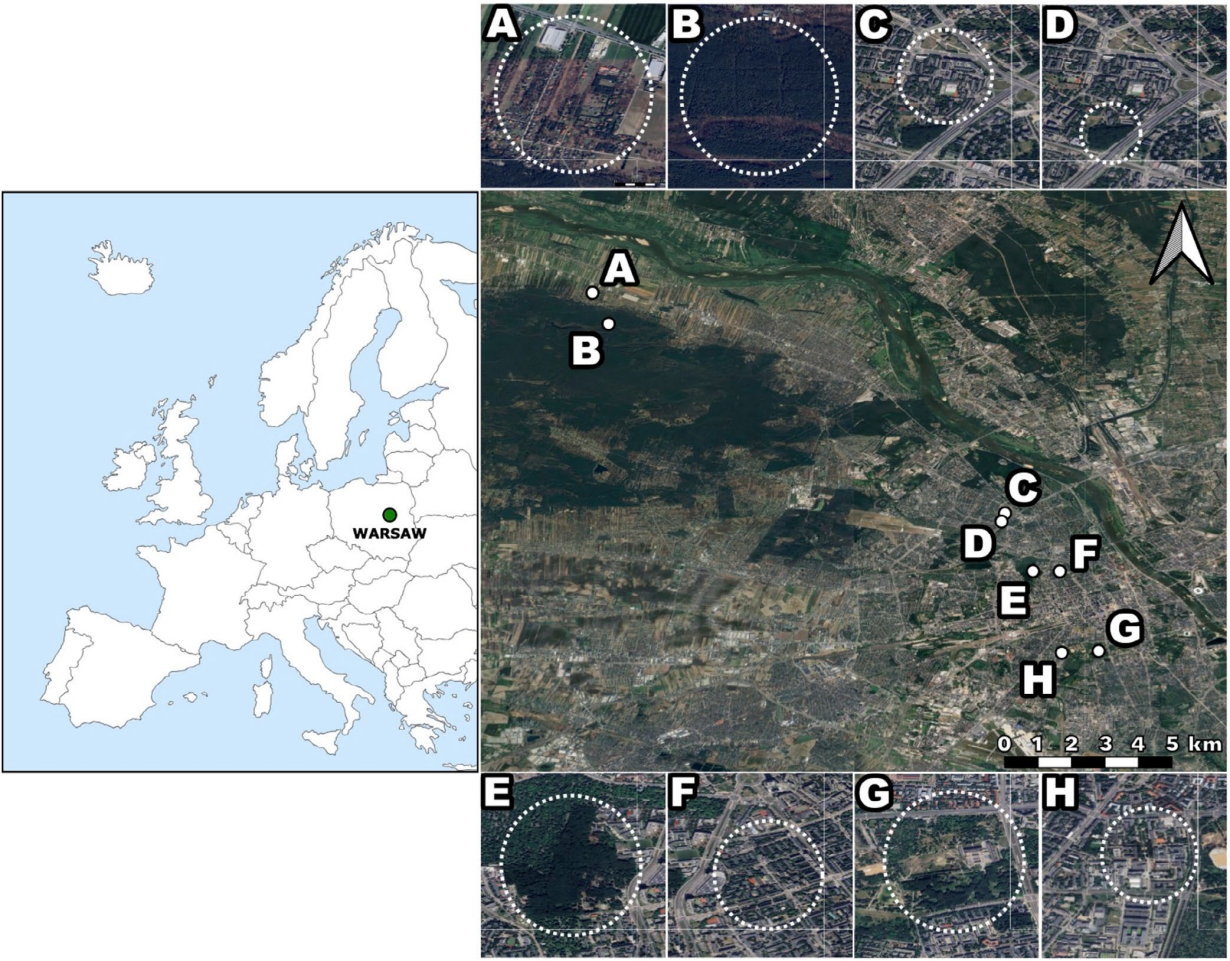
Map of study sites located in a gradient of urbanisation in the capital city of Warsaw, Poland. These include: A suburban village (A), a national park (B), two residential areas (C, F), two urban woodlands (D, E), an urban park (G) and an office area (H). Satellite images acquired from Google Maps (2024).

This study is part of a long‐term research project investigating the ecology and evolution of passerine birds within an urban mosaic. Initiated in 2016, the project consists of 500 woodcrete nestboxes (Schwegler, type 1b with 32 mm entrance) arranged on a 50 m grid across eight study sites in the Capital City of Warsaw, Poland. Each site varies in urbanisation levels, and the setting consists of a suburban village, a national park, two residential areas, two urban woodlands, an urban park and an office area (Figure 1). For this study, data were collected over two breeding seasons, in 2021 and 2022. Further descriptions of each study site can be found in the Supporting Information (Text S1).

### 2.2 Quantifying Urbanisation

The definition of urbanisation is not agreed upon unanimously among researchers (Szulkin et al. 2020). Urban areas are increasingly quantified using continuous parameters, such as land‐cover classes, specific environmental variables or pollution gradients, which allows for finer inference than qualitative urban–rural assessments (Szulkin et al. 2020). Here, urbanisation was quantified using the amount of Impervious Surface Area (ISA) as a proxy—that is all built‐up areas, such as infrastructural networks and buildings, around the nestbox vicinity. ISA is a reliable and repeatable indicator of urbanisation worldwide (Szulkin et al. 2020; Murray‐Stoker et al. 2025). ISA also covaries positively with other urban features, such as temperature, sound or noise pollution, and negatively covaries with NDVI (Normalised Difference Vegetation Index, a measure of vegetation greenness) and tree cover (Szulkin et al. 2020). The percentage of ISA was measured in a 100 m radius for each nestbox using the open source software QGIS (version 3.40), with a 20‐m‐pixel resolution extrapolated via satellite imagery from 2015 (Copernicus Land Monitoring Services, https://land.copernicus.eu/sitemap) as described in Szulkin et al. (2020). The 100 m radius also corresponds to a conservative estimate of distance typically flown from the nest by great tits to forage for food for their chicks in urban settings (Seress et al. 2025).

### 2.3 Life History Data Collection and Bird Measurements

Starting from the last week of March, each nestbox across the study sites was visited at weekly intervals to monitor its occupancy and the following breeding stages. Once incubation started, hatching date was estimated (12 days from clutch completion) (Álvarez and Barba 2014). Nestboxes were visited on the expected hatching date (±1 day) and every other day thereafter until hatching (hatching day = day 1) or until the nest was considered deserted. Each adult bird was caught at the nestbox once between 10 and 15 days after hatching, either with traps or by manually blocking the nestbox entrance. All birds were ringed using standard‐numbered metal rings supplied by the Polish Ringing Centre (Museum and Institute of Zoology, Polish Academy of Sciences). All retraps from previous years were noted. Adult biometric measurements were collected as follows: birds were sexed and aged as first year breeders or older (two age levels) according to plumage characteristics (Demongin 2016), wing length was measured to the nearest 1.0 mm with a wing‐ruler, tarsus to the nearest 0.1 mm with a manual calliper, and weight was recorded to the nearest 0.1 g with a digital scale. In this study, only data on great tit first broods, defined as clutches that started within 30 days from the recording of the first egg at a given site and year (Van Balen 1973), were included.

### 2.4 Egg Photography

**FIGURE 2:**
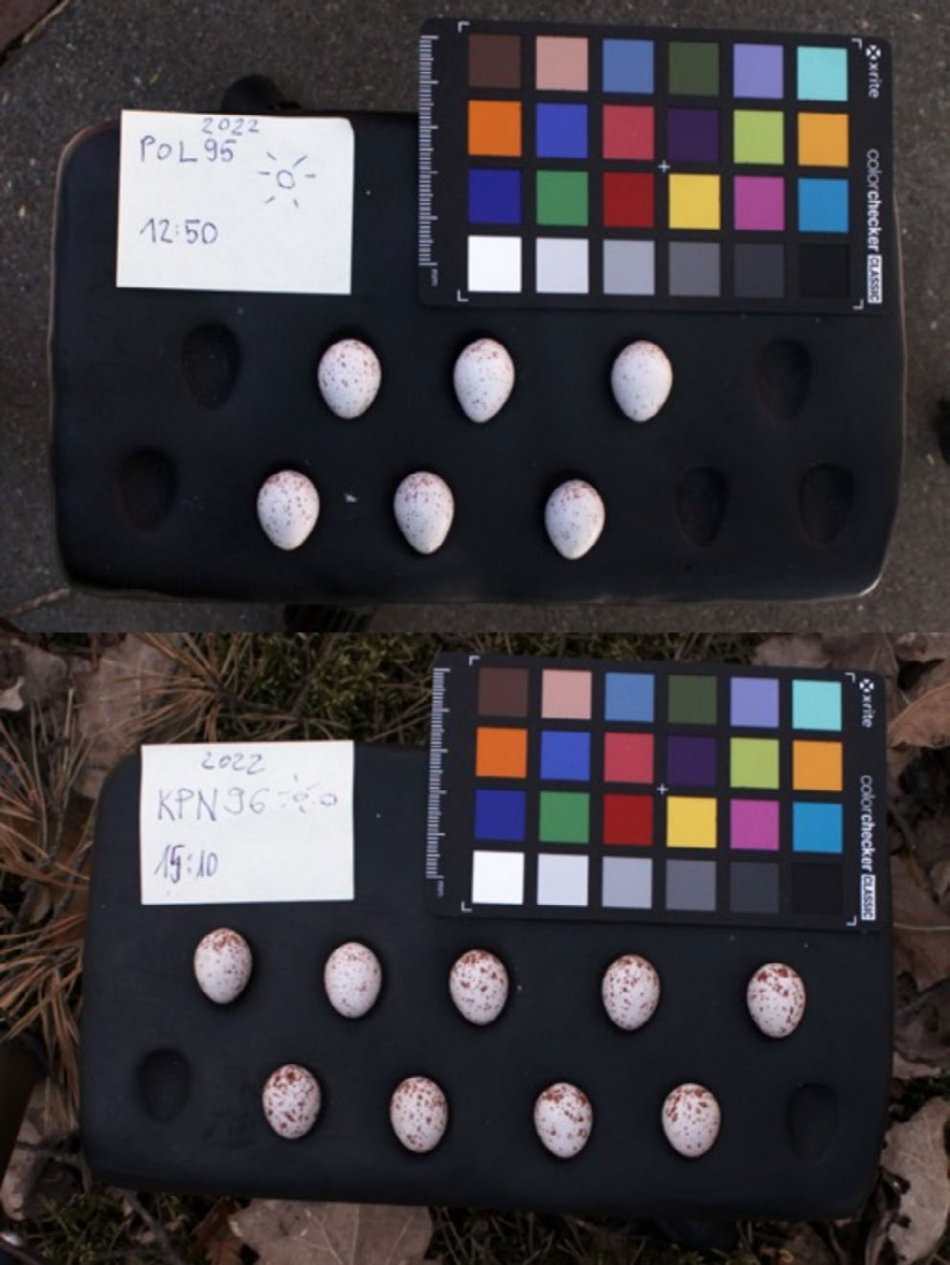
Two great tit clutches differing in pigmentation patterns. Top line—a lightly pigmented clutch with mostly medium and small sized spots, laid in an *urban park*. Bottom line—a heavily pigmented clutch with a lot of large spots laid in a *natural forest*.

To collect data on egg characteristics, clutches were photographed during the incubation phase, approximately 10 days after clutch completion. Egg pictures were taken using a Canon EOS 1100D reflex camera with a Canon EF‐S 18–55 mm f/3.5–5.6 IS II lens, set on a tripod. Eggs were put in shallow pits on a custom‐made tray. The tray was fixed to a small tripod and equipped with grey colour standards (X‐Rite ColorChecker Classic Mini, X‐Rite, Grand Rapids, USA) and a scale bar (5 cm with 1 mm divisions) (Figure 2). Using a round spirit level, both the camera and the tray were aligned perpendicularly to the ground. All clutches were photographed between 9:00 AM and 4:15 PM, when the sun was not covered by clouds. To avoid overexposure, equipment was set with a photographer casting a shadow on the whole tray with eggs (following Troscianko et al. 2016). Aperture f/8, ISO 400 and focal length of 18 mm were used. Only shutter speed was manipulated to adjust the duration of light exposure. Exposure bracketing with ±1 step was used. All images were saved in RAW format. To generate a full record of egg speckling, eggs were first photographed from one side, then turned 180° sideway and photographed again. Furthermore, eight clutches (61 eggs) were photographed on two separate days to check for data collection repeatability. Eggs from clutches that were to be photographed again were individually numbered, so that they could be identified later. To test for a potential effect of the placement of the eggs on the tray on the measurements, eggs were arranged differently (i.e., put in different pits) between the trials and the order was recorded for further repeatability estimates.

### 2.5 Digital Imaging

Pictures were processed using the ImageJ software (version 1.54d) with MICA Toolbox plug‐in designed for research in the field of visual ecology (Troscianko and Stevens 2015a). First, using the Photo Screening tool, the brightest and simultaneously not over‐exposed images were selected for both sides of the eggs for all clutches. Second, images were calibrated with respect to the darkest and the brightest grey standards (3.22% and 90.03% of light reflectance respectively) and a 10 mm scale bar was selected to set the scale.

Using the multipoint tool, each egg was outlined using at least eight anchor points along the egg's edges, which also provided data on egg *volume* (Troscianko 2014). Afterwards, the Scale Bar Calculator tool was used to obtain the minimum px/mm factor for the set of pictures (10.7012 px/mm rounded down to 10.5 px/mm). This factor is needed to make pattern measurements comparable between images and should always be rounded down (Troscianko and Stevens 2015b). Finally, colour and pattern were automatically measured using custom‐written scripts for the MICA Toolbox that segmented the egg image into spots and eggshell background (Szala and Šulc, in prep). The following settings were used for the segmentation: 50 px radius for thresholding (Phansalkar et al. 2011), a difference of 128 px Gaussian blur (Gómez et al. 2018) and the outline was shrunk by 10% of egg width to avoid measuring shadows occurring at the egg's edges. Egg spot colour and the extent to which spots cover the egg area (in %) were shown to best correlate with the concentration of protoporphyrin in great tit eggshells (Wegmann et al. 2015; Hargitai, Nagy, Herényi, et al. 2016). For this reason, these two features of eggshell coloration were measured: spot colour was calculated as the reflectance in the red channel divided by the sum of reflectance in red, green and blue channels (*spot red chroma* hereafter) and *spot percentage* as the eggshell area covered by spots divided by the whole surface area of the egg side.

After normalisation, a significant portion of the eggs (*N* = 320) were identified as overexposed near the pointed end (‘top’). To retain as large a sample size as reliably possible and exclude overexposed regions, measurements were restricted to a sub‐area of eggs. This sub‐area comprised 85% of every egg's width, 30% of its length and was positioned at the 0.6 of its length (with 0 being the ‘top’ and 1 being the ‘bottom’ edge of the egg) and constituted c. 27% (minimum = 26.02%, median = 26.82%, maximum = 27.87%) of one side of the egg surface. Overexposed pixels were counted in the sub‐area and eggs with > 0.5% of overexposed pixels were excluded from further analyses (*N* = 29). Using pictures of clutches that were not overexposed (*N* = 382 eggs from 46 clutches), the repeatability of pigmentation characteristics between the whole egg surface and its sub‐area was tested. Given good repeatability (*R* > 0.82; see Section 3), measurements of eggshell pigmentation (*spot red chroma* and *spot percentage*) based on the sub‐area of eggs were used in further analyses.

Data from clutches in which no egg was overexposed from either side (*N* = 359 eggs from 43 clutches) were extracted first and repeatability tests of egg traits between two sides were performed (see Section 2.7). The repeatability of egg volume was above 0.9 and the repeatabilities of pigmentation characteristics were above 0.8 (see Section 3). Therefore, the rest of the clutches were imaged using just one picture (side) instead of two, and data derived from only one side of each egg were used in the models.

### 2.6 Visual Scoring of Egg Pigmentation

To retain comparability with earlier studies on egg pigmentation, a method of visual egg pigmentation scoring, following Gosler et al. (2000) was also applied. Each egg was visually scored in three categories: pigment *intensity* (scored in 0.5 increments from 1 for palest to 5 for the darkest), *distribution* (scored in 0.5 increments from 1 for > 90% of spots concentrated at one end to 5 for an evenly distributed), and *spot‐size* (scored in 0.5 increments from 1 for small spots to 3 for large spots). All eggs were scored by the same observer, with the scores being assigned using pictures of both sides of the egg. To test for within‐observer repeatability of scoring, the observer assessed one egg from each brood again, in a randomised order. To learn more about different methods of assessing eggshell pigmentation and their limitations, see Brulez et al. (2014) and Stevens (2011).

### 2.7 Statistical Analysis

All statistical analyses were performed in R (version 4.1.2). To test for repeatability of egg *volume* and pigmentation between (1) sides of eggs, (2) trials (i.e., same clutches photographed twice on different days), (3) two visual scoring sessions and (4) whole eggshell side and sub‐area, the *rpt* function in the *rptR* package (version 0.9.22, Stoffel et al. 2016, 2017) was used. *Egg ID* was used as a grouping factor for all tests. The confidence interval was set to 0.95, number of bootstrap samples to 500, number of permutations to 0 and Gaussian distribution was used. Repeatability reliability categories, based on *R* value, were assigned following Koo and Li (2016).

**FIGURE 3:**
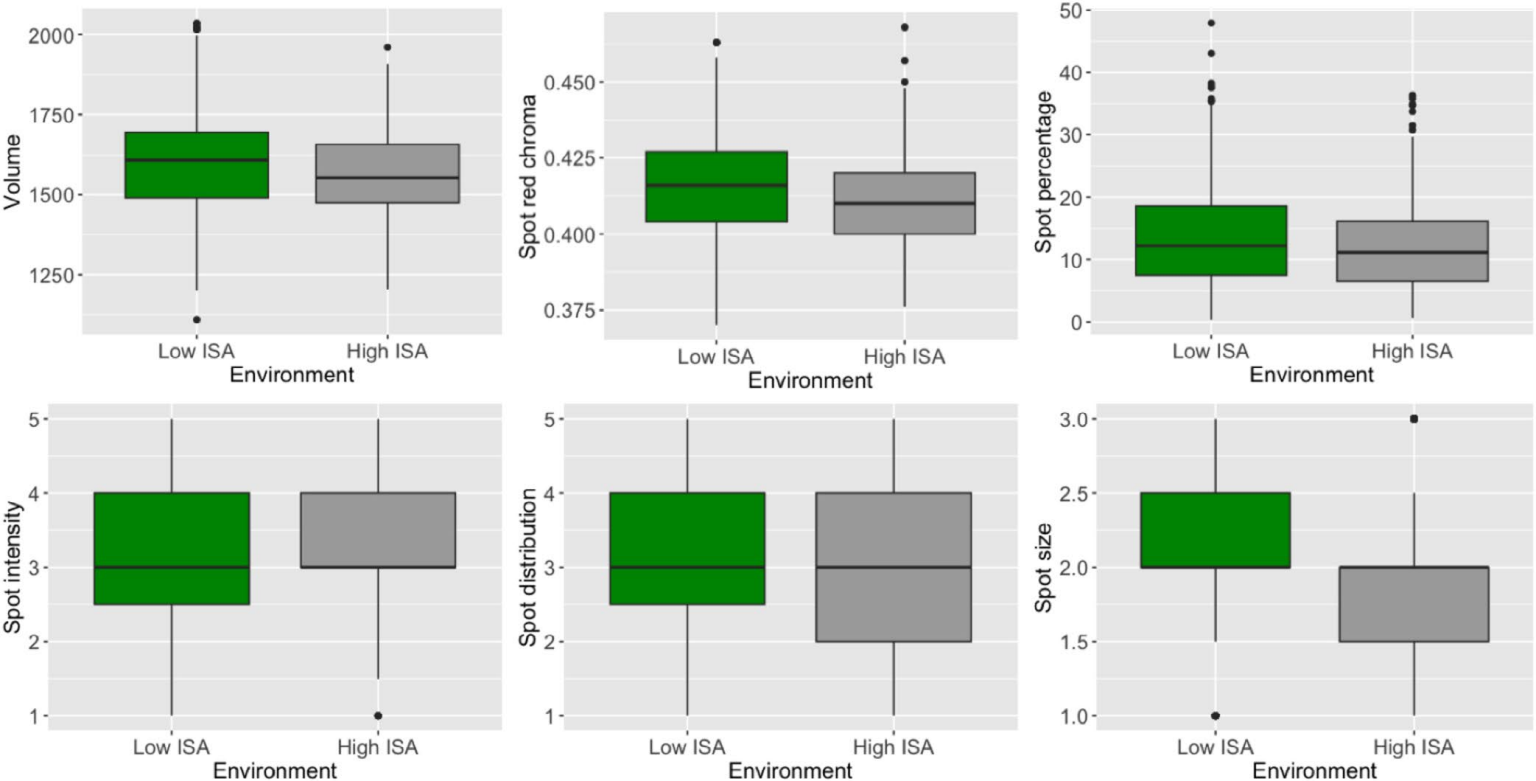
Comparison of six great tit egg traits between *Low ISA* and *High ISA* environments, when using digital quantification (top line) and visual scoring (bottom line); *N*_eggs_ = 568. No significant effect of urbanisation was noted in either digital quantification of trait variation (egg *volume*, *spot red chroma*, *spot percentage*; see Table 2, Table S2 for models) or visual scoring (egg spot *intensity*, *distribution* or *size*; see Table S2 for models).

To test for an association between egg traits (egg *volume* and pigmentation characteristics: *spot red chroma*, *percentage*, *intensity*, *distribution* and *size*), urbanisation and life‐history traits, Generalised Linear Mixed Models (GLMMs) were fitted using Template Model Builder (glmmTMB) (Brooks et al. 2017). For continuous predictors, mean‐centered values of *ISA* (%), *lay date* (date of the first egg laid in the clutch; starting from 1st of April recorded as 1), *clutch size* and *female body condition* (calculated following Peig and Green 2009) were used. In terms of categorical variables, *year* was fitted as a fixed effect with two levels. To avoid pseudoreplication, *clutch ID*, *site ID* and *female ID* were included as random effects, accounting for eggs sampled within the same clutch and site and for females sampled in both years, respectively. To detect potential multicollinearity issues, the *check_collinearity* function was used (*performance* package; Fox and Weisberg 2019): all VIF (variance inflation factor) values were below 2 and therefore no continuous predictors were excluded. To verify model fits and assumptions, the *DHARMAa* package was used (see Figure S1) (Hartig 2022). Data were visualised using *ggplot2* (Wickham 2011). Environmental categories used in Figure 3 were defined based on the median value of ISA for all clutches, that is, *Low ISA*≤median, *High ISA* > median (see Section 3 for details).

## 3 Results

### 3.1 Sample Size and Impervious Surface Area

Following digital processing, the final dataset included measurements from 718 eggs from 90 broods. Final GLMMs included 568 eggs from 74 broods as data were unavailable for 16 females that were not caught or escaped before all measurements were performed. The amount of Impervious Surface Area ISA (in %) in a 100 m radius around each clutch used in this study varied from a minimum of 0.00% to a maximum of 60.73%, with an average at 12.73% and median at 4.73%. For details see Table S1.

### 3.2 Repeatability of Egg Traits

The results of repeatability (*R*) tests summarised in Table 1 were categorised following Koo and Li (2016), that is, *R* < 0.5: poor; 0.5 ≤ *R* < 0.75: moderate; 0.75 ≤ *R* < 0.9: good, *R* ≥ 0.9: excellent. Egg traits were found to have excellent (*volume*) or good (*spot red chroma* and *percentage*) repeatability between egg sides (Table 1). Importantly, the reliability (defined as consistency of results when measurements are repeated under similar conditions) of both digital and visual quantification methods were confirmed with good repeatability (Table 1). Both *spot red chroma* and *percentage* had good repeatability between the whole side of an egg and its sub‐area (Table 1), which allowed for inclusion of eggs that were partially overexposed in the analysis (for details, see Section 2.5).

**TABLE 1:** Egg traits repeatability in terms of trait quantification and data collection methods: Between (1) two sides of eggs, (2) two trials (i.e., same clutches photographed twice on two different days), (3) two visual scoring sessions and (4) a whole eggshell side and its sub‐area.

	*R*	SE	CI	*p*
**Egg sides** ***N* = 359 × 2**				
Volume	0.98	0.00	[0.98, 0.98]	< 0.001***
Spot red chroma	0.83	0.02	[0.79, 0.85]	< 0.001***
Spot percentage	0.83	0.02	[0.79, 0.86]	< 0.001***
**Trials** ***N* = 61 × 2**				
Volume	0.79	0.05	[0.67, 0.87]	< 0.001***
Spot red chroma	0.78	0.05	[0.66, 0.86]	< 0.001***
Spot percentage	0.83	0.04	[0.73, 0.89]	< 0.001***
**Visual scoring** ***N* = 90 × 2**				
Intensity	0.88	0.03	[0.82, 0.92]	< 0.001***
Distribution	0.87	0.03	[0.80, 0.91]	< 0.001***
Spot size	0.79	0.04	[0.70, 0.86]	< 0.001***
**Sub‐area** ***N* = 382 × 2**				
Spot red chroma	0.86	0.02	[0.79, 0.85]	< 0.001***
Spot percentage	0.83	0.02	[0.79, 0.86]	< 0.001***

*** *p* < 0.001.

### 3.3 Covariation Between Urbanisation, Egg Traits and Life‐History Traits

Urbanisation modelled as the amount of *ISA* in nest vicinity was not associated with egg *volume* (Table 2) or egg pigmentation traits (*spot red chroma*, *spot percentage*) as quantified with digital imaging (Figure 3, top line; Table S2). In contrast to a lack of urban‐driven effects on egg traits, egg *volume* covaried positively with *lay date* and *female body condition* (Table 1). Egg traits also varied between years: eggs in 2022 were larger (estimate ± SD = 90.84 mm^3^ ± 24.19 mm^3^) and less covered in spots (estimate ± SD = 7.66% ± 1.18%) than in 2021 (Table 1, Table S2). Finally, *spot red chroma* was not associated with any of the examined explanatory variables (Table S2).

**TABLE 2:** GLMMs with Gaussian distribution testing the association between egg *volume*, urbanisation and life‐history traits, *N*_eggs_ = 568. Continuous predictors were mean‐centered (mc).

Family: Gaussian, Random = Site ID (*N* = 8) + Clutch ID (*N* = 74) + Female ID (*N* = 66)				
Model structure (glmmTMB): Volume ~ ISA_mc_ + Lay date_mc_ + Clutch size_mc_ + Female body condition_mc_ + Year				
Volume	Estimate	SE	*z*‐value	*p*
Intercept	1536.07	29.17	52.76	< 0.001***
ISA	−0.25	1.02	−0.25	0.804
Lay date	6.72	2.82	2.38	0.017*
Clutch size	5.51	9.40	0.59	0.558
Female body condition	32.65	15.10	2.16	0.03*
Year	90.84	24.19	3.76	< 0.001***

* *p* < 0.05.

*** *p* < 0.001.

Consistently with models using egg traits data quantified with digital imaging, models using data based on visual egg pigmentation scoring (*spot intensity, distribution and size*) were not influenced by urbanisation (*ISA*) (Figure 3, bottom line; Table S2). The only association found in all three models was a negative one, between *female body condition* and *spot intensity* (Table S2).

## 4 Discussion

Based on a 2‐year data set containing 568 eggs from 74 clutches, spread across 8 study sites differing in urbanisation levels, this study reports that while great tit egg *volume* and pigmentation patterns covary with life history variables (i.e., *lay date*, *female body condition* and *year*), these egg traits are not affected by urbanisation. Although literature for egg trait variation is available for rural habitats (see Section 1), knowledge about urban‐driven variation is limited (Hõrak et al. 1995; Bailly et al. 2016; Hargitai, Nagy, Nyiri, et al. 2016; Bańbura et al. 2010). Moreover, to the best of the authors' knowledge, this is the first study on egg trait variation that incorporates a fine‐scale spatial quantification of urbanisation in the analyses: in this study, all models included the amount of impervious surface area in the nest surroundings, as opposed to using dichotomical approach (rural vs. urban), commonly used in urban ecology and evolution research. While analysing egg trait data, both digital and visual quantification methods were applied in parallel. To confirm that the methods of data collection and analysis were reliable, four repeatability tests regarding different aspects of the methodology were conducted, providing a reliable quantification of egg trait variation (Table 1). Therefore, this study offers valuable insight into the scope for detecting the potential effect of urbanisation on eggs, a key aspect of bird reproduction.

Based on selected literature, Table 3 presents a summary of findings regarding egg size and pigmentation and associated factors in great tits and blue tits. What emerges from the current study and earlier research is the strong heterogeneity in variables associated with egg traits between studies. Thus, while some studies report specific associations, other studies did not report the same biological signals. Moreover, within the same study, the presence of some signals depended on explanatory variables used in the specific model (Table 3; Hargitai, Nagy, Herényi, et al. 2016; Sanz and García‐Navas 2009). Such inconsistencies in earlier research outcomes on egg size have been highlighted by Encabo et al. (2001), as different trends could be found in the same population in different years of study. Therefore, if possible, research on egg traits should rely on large samples, spanning multiple years. Furthermore, obtained results should be interpreted cautiously.

**TABLE 3:** Summary of associations between great tit (GT) and blue tit (BT) egg traits and explanatory variables reported in selected literature. Associations (or lack thereof) as tested in this study are indicated in bold. As some studies used either egg volume or mass (which are highly correlated, *R* = 0.97, Van Noordwijk et al. 1981) the collective category *egg size* was used. *Female condition* refers to *body mass*, *tarsus length*, or *body condition*.

	Egg size	Spot red chroma	Spot percentage	Spot intensity	Spot distribution	Spot size
Lay date	**Positive**: **This study (GT)** Wilkin et al. 2009 (GT) Negative: Pitt et al. 2024 (BT) No trend: Eeva and Lehikoinen 1995 (GT) Encabo et al. 2001 (GT) Hargitai, Herényi, Nagy, et al. 2016 (GT) Järvinen and Pryl 1989 (GT) Szigeti et al. 2007 (BT)	Positive: Negative: **No trend:** **This study (GT)**	Positive: Negative: Hargitai, Herényi, Nagy, et al. 2016 (GT) **No trend**: **This study (GT)** Martínez‐de La Puente et al. 2007 (BT)	Positive: Negative: Gosler et al. 2000 (GT) Hargitai, Herényi, Nagy, et al. 2016 (GT) **No trend**: **This study (GT)** Sanz and García‐Navas 2009 (BT)	Positive: Gosler et al. 2000 (GT) Negative: **No trend**: **This study (GT)** Hargitai, Herényi, Nagy, et al. 2016 (GT) Sanz and García‐Navas 2009 (BT)	Positive: Negative: Gosler et al. 2000 (GT) **No trend**: **This study (GT)** Hargitai, Herényi, Nagy, et al. 2016 (GT)
Clutch size	Positive: Encabo et al. 2001 (GT) Negative: Bańbura et al. 2018 (BT) You et al. 2009 (GT) **No trend:** **This study (GT)** Eeva and Lehikoinen 1995 (GT) Järvinen and Pryl 1989 (GT)	Positive: Negative: Hargitai, Nagy, Herényi, et al. 2016 (GT) **No trend**: **This study (GT)** Malinowska et al. 2023 (GT)	Positive: Malinowska et al. 2023 (GT) Negative: **No trend**: **This study (GT)** Hargitai, Nagy, Herényi, et al. 2016 (GT) Martínez‐de La Puente et al. 2007 (BT)	Positive: Negative: **No trend:** **This study (GT)** Gosler et al. 2000 (GT) Hargitai, Nagy, Herényi, et al. 2016 (GT) Sanz and García‐Navas 2009 (BT)	Positive: Sanz and García‐Navas 2009 (BT) Negative: **No trend:** **This study (GT)** Gosler et al. 2000 (GT) Hargitai, Nagy, Herényi, et al. 2016 (GT)	Positive: Negative: **No trend:** **This study (GT)** Gosler et al. 2000 (GT) Hargitai, Nagy, Herényi, et al. 2016 (GT)
Female condition	**Positive**: **This study (GT)** Järvinen and Pryl 1989 (GT) Ojanen et al. 1979 (GT) Negative: No trend: Szigeti et al. 2007 (BT)	Positive: Negative: **No trend:** **This study (GT)** Hargitai, Nagy, Herényi, et al. 2016 (GT)	Positive: Negative: Martínez‐de La Puente et al. 2007 (BT) **No trend**: **This study (GT)** Hargitai, Nagy, Herényi, et al. 2016 (GT)	Positive: Sanz and García‐Navas 2009 (BT, tarsus length) **Negative:** **This study (GT)** No trend: Hargitai, Nagy, Herényi, et al. 2016 (GT) Sanz and García‐Navas 2009 (BT, body mass)	Positive: Negative: **No trend**: **This study (GT)** Hargitai, Nagy, Herényi, et al. 2016 (GT) Sanz and García‐Navas 2009 (BT)	Positive: Negative: **No trend:** **This study (GT)** Hargitai, Nagy, Herényi, et al. 2016 (GT)
Year effect	**Present**: **This study (GT)** Encabo et al. 2001 (GT) Szigeti et al. 2007 (BT) Not present: Bańbura et al. 2018 Eeva and Lehikoinen 1995 (GT) Järvinen and Pryl 1989 (GT)	Present: **Not present**: **This study (GT)**	**Present**: **This study (GT)** Hargitai, Nagy, Herényi, et al. 2016 (GT, model 2) Not present: Hargitai, Nagy, Herényi, et al. 2016 (GT, model 1)	Present: Gosler et al. 2000 (GT) Hargitai, Nagy, Herényi, et al. 2016 (GT, model 1) **Not present**: **This study (GT)** Hargitai, Nagy, Herényi, et al. 2016 (GT, model 2) Sanz and García‐Navas 2009 (BT)	Present: Gosler et al. 2000 (GT) Hargitai, Nagy, Herényi, et al. 2016 (GT, model 2) **Not present:** **This study (GT)** Hargitai, Nagy, Herényi, et al. 2016 (GT, model 1) Sanz and García‐Navas 2009 (BT)	Present: Gosler et al. 2000 (GT) **Not present:** **This study (GT)** Hargitai, Nagy, Herényi, et al. 2016 (GT, model 1&2)

None of the investigated egg traits were associated with urbanisation. This aligns with previous studies conducted on great tits, reporting that neither egg size nor pigmentation was associated with urban habitat (Hõrak et al. 1995; Bańbura et al. 2010; Bailly et al. 2016; Hargitai, Nagy, Nyiri, et al. 2016). Moreover, two studies incorporated direct eggshell thickness measurements and also reported no differences between urban and rural habitats (Bailly et al. 2016), or only marginal differences (Hargitai, Herényi, Nagy, et al. 2016; *F*_1,35_ = 4.53, *p* = 0.040; mean ± SD: woodland: 79.1 ± 3.0 μm; urban: 81.6 ± 3.8 μm).

In contrast, other studies reported urban‐driven differences in blue tit egg size, with larger eggs found in urban habitats than in rural habitats (Bailly et al. 2016; Bańbura et al. 2010). This species‐specific egg trait variation in response to urbanisation may be driven by differing dietary and foraging responses of the two passerines. A recent study by Chatelain et al. (2026), showed that great tit diet was less diverse in urban areas and included more energy‐dense (e.g., sunflower seeds) rather than nutrient‐rich (e.g., arthropods) items, while no such trend was found in blue tits. Moreover, Mackenzie et al. (2014) found that unlike great tits, urban blue tits showed strong preference to forage in native vegetation, which better supports snails than non‐native plants (Kappes et al. 2007). Thus, it is possible that compared to great tits, blue tits cope better with nutrient acquisition in the urban environment during egg laying. Interestingly, it is not uncommon for these two passerine species to exhibit distinct responses to urbanisation, other such traits include: yolk carotenoid concentration (Bailly et al. 2016), plumage colouration (Janas et al. 2026), timing of breeding (Solonen and Hildén 2014; Branston et al. 2021), extra‐pair paternity (Di Lecce et al. 2024), response to novel objects (Corsini et al. 2022), and genetic differentiation (Markowski et al. 2021), indicating a limited capacity for urban ecological research generalisation, even among closely related species.

Results from this and earlier studies convincingly suggest that great tit eggs do not differ in terms of size and pigmentation patterns between urban and rural environments. Given that both egg size and pigmentation patterns were previously proposed as clutch quality indicators as they are associated with hatching success (Sanz and García‐Navas 2009; Krist 2011), this study shows that urbanisation does not necessarily affect egg quality in great tits. Indeed, research conducted on the same population as this study revealed that there are no urban‐driven differences in hatching success or in chick mass at hatching (Corsini et al. 2020; Corsini and Szulkin 2025; Table S3). However, Bailly et al. (2016) and Charmantier et al. (2017), did find a slightly lower hatching success in cities. Importantly, urban clutches have consistently fewer eggs, a trend that was reported both in this population (Corsini and Szulkin 2025; Supporting Information), as well as in others (see meta‐analysis by Chamberlain et al. 2009).

A strong argument that urban females are environmentally constrained by nutrient availability was suggested by Pitt et al. (2024). An egg removal experiment conducted on blue tits revealed that in comparison to forest birds, urban birds have a lower capacity to lay replacement eggs (i.e., approximately 2 vs. 0.36 new eggs laid in forest vs. urban environments). Therefore, it is likely that birds in urban environments have an ability to cope with limited food and nutrient availability while laying the eggs, but only to some extent—which is translated into eggs of similar characteristics, but with a smaller number of them in each clutch.

In terms of coping mechanisms, it is likely that calcium‐seeking urban females either increase their search efforts for calcium and/or that they supplement their diet with other resources. In calcium‐poor natural forests, great tits nesting near human settlements were compensating for the lack of snails with anthropogenic resources such as chicken eggshells (Graveland 1996), while some other suggestions include plaster, ash and calcium leaching from concrete (Eeva and Lehikoinen 2004), as well as grit (Graveland and Berends 1997). Moreover, urban tits are known to have higher exploration rates (Charmantier et al. 2017) and to travel further while seeking food for their chicks (Jarrett et al. 2020; Seress et al. 2025). At the same time, females that were experimentally deprived of calcium were seeking it actively, doubling the time spent on searching and resorting to eating sand, small stones or even their own eggs (Graveland and Berends 1997). While it could be expected that in urban areas, a smaller clutch size would act as a coping response to limited food availability (Seress et al. 2020) and counteract mortality rates during later stages of chick development, no such effect was observed: ultimately, an increased amount of impervious surfaces consistently results in fewer fledglings leaving the nest (Charmantier et al. 2017; Corsini and Szulkin 2025), meaning that urban females still lay more eggs than they can successfully rear (Pitt et al. 2024).

## 5 Conclusions

This study highlights the complexities of urban phenotypic egg trait variation. While it was expected that urbanisation would affect egg traits negatively, as such trends were reported in calcium‐poor natural habitats, and for other avian reproductive traits in cities, no such pattern was found in this study, despite using a large dataset and fine‐scale quantification of urban habitat. Instead, urban birds lay eggs that are similar to those found in more natural habitats in terms of *volume* and pigmentation patterns, but often have fewer of them in a clutch. Given the environmental constraints arising in the urban habitat and associated, consistently lower fledging success, investing additional resources into larger clutches is not likely to benefit their reproduction and could instead further hinder it.

Although this paper broadens our understanding of egg trait variation in the urban space, we acknowledge that it lacks direct quantification of environmental calcium availability. Therefore, we recommend that future studies on the topic incorporate soil or food‐item (both natural and anthropogenic) analysis, or an experimental approach with calcium supplementation in urban and natural populations. Such studies could confirm the mechanistic links underlying the findings presented in this article. Moreover, including additional parameters, such as egg trait variation associated with laying sequence (shells tend to change in size and pigmentation throughout laying; You et al. 2009) would also be of value. Lastly, future long‐term research on other avian species, and in cities with different socio‐geographical settings, is needed as factors associated with eggshell traits are highly context‐specific (Table 3).

## Author Contributions

**Ignacy Stadnicki:** conceptualization (lead), data curation (equal), formal analysis (lead), funding acquisition (supporting), methodology (equal), visualization (lead), writing – original draft (lead), writing – review and editing (equal). **Michela Corsini:** conceptualization (supporting), data curation (equal), formal analysis (supporting), funding acquisition (supporting), supervision (equal), writing – review and editing (equal). **Klaudia Szala:** methodology (equal), software (lead), supervision (supporting), writing – review and editing (equal). **Andrew Gosler:** methodology (equal), supervision (equal), writing – review and editing (equal). **Marta Szulkin:** funding acquisition (lead), project administration (lead), resources (lead), supervision (lead), validation (lead), writing – review and editing (equal).

## Funding

This work was supported by Uniwersytet Warszawski (501‐D114‐20‐0004410) and Narodowe Centrum Nauki (2014/14/E/NZ8/00386, 2017/25/N/NZ8/02852 and 2021/41/B/NZ8/04472).

## Ethics Statement

This research was carried out with a permit from the Regional Directorate for Environmental Protection in Warsaw, Poland. Permissions for bird ringing were granted by the Polish Ringing Centre (Museum and Institute of Zoology, Polish Academy of Sciences).

## Conflicts of Interest

The authors declare no conflicts of interest.

## Supporting information


**Figure S1:** DHARMa diagnostics of models testing for the association between egg traits, urbanisation and life‐history traits.
**Table S1:** Summary of ISA values for clutches from all study sites.
**Table S2:** GLMMs with Gaussian distribution testing the association between egg pigmentation traits, urbanisation and life‐history traits.
**Table S3:** Linear Mixed Effect Models (LMMs) testing the association between Impervious Surface Areas (%) measured in a 100 m radius around each nestbox, and number of hatched offspring in great tits and blue tits.
**Text S1:** Descriptions of study sites set in a gradient of urbanisation in Warsaw, Poland.

## Data Availability

Data and codes for this manuscript are available at Mendeley Data Repository: https://data.mendeley.com/datasets/kyjggytnpw/1 (https://doi.org/10.17632/kyjggytnpw.1).
