# Supplementary material for: An Analysis of Great Tit Egg Traits Across the City Mosaic: Urbanisation Does Not Affect Egg Size and Pigmentation Patterns
Source: Ecol Evol. 2026 Jul 30;16(8):e73867. doi: 10.1002/ece3.73867 (PMC13421803; doi:10.1002/ece3.73867)
Supplement: Supplementary file 1 — Figure S1: DHARMa diagnostics of models testing for the association between egg traits, urbanisation and life‐history traits. Table S1: Summary of ISA values for clutches from all study sites. Table S2: GLMMs with Gaussian distribution testing the association between egg pigmentation traits, urbanisation and life‐history traits. Table S3: Linear Mixed Effect Models (LMMs) testing the association between Impervious Surface Areas (%) measured in a 100 m radius around each nestbox, and number of hatched offspring in great tits and blue tits. Text S1: Descriptions of study sites set in a gradient of urbanisation in Warsaw, Poland. [file ECE3-16-e73867-s001.docx]

**SUPPORTING INFORMATION**

**Title:**

An Analysis Of Great Tit Egg Traits Across The City Mosaic: Urbanisation Does Not Affect Egg Size And Pigmentation Patterns

**Authors:**

Ignacy Stadnicki^a*,f,,1^, Michela Corsini^b,2^, Klaudia Szala^c,d,3^, Andrew Gosler^e,f,4^ & Marta Szulkin^a,5^

**Affiliations:**

*^a^Institute of Evolutionary Biology, Biological and Chemical Research Centre, Faculty of Biology, University of Warsaw, Warsaw, Masovian Voivodeship, Poland*

*^b^Institute for Alpine Environment, Eurac Research, Viale Druso 1, Bolzano, Italy*

*^c^Department of Avian Biology and Ecology, Faculty of Biology, Adam Mickiewicz University, Poznań, Poland*

*^d^Department of Biology, George Mason University, Fairfax, VA, USA*

*^e^Edward Grey Institute of Field Ornithology, Department of Biology, University of Oxford, Oxford, United Kingdom*

*^f^Institute of Human Sciences, School of Anthropology & Museum of Ethnography, University of Oxford, Oxford, United Kingdom*

**ORCID:**

*^1^ 0000-0002-0526-9610*

*^2^ 0000-0001-5196-086X*

*^3^ 0000-0003-1697-2149*

*^4^ 0000-0002-8074-8064*

*^5^ 0000-0002-7355-5846*

**Corresponding author email:**

[i.stadnicki@student.uw.edu.pl](mailto:i.stadnicki@student.uw.edu.pl)

**ABSTRACT**

1. Rapid urbanisation provides remarkable opportunities to study how sudden, extreme changes impact wildlife. Compared to natural areas, cities are characterised by factors affecting abiotic (e.g., climate, habitat fragmentation) and biotic (e.g., species composition, phenology) components of the ecosystem, ultimately changing the ecological and evolutionary dynamics of those habitats. Similarly to many other taxonomic groups, urban birds differ from rural birds in morphology, behaviour and reproductive patterns. Yet potential associations between urbanisation and avian egg traits a key aspect of birds life-cycle — remain under-researched. Given the limited availability of primary natural calcium sources (snails) in cities, eggs from heavily urbanised areas were expected to be smaller and more pigmented, indicating thinner shells and lower overall egg quality.
2. To better understand how urbanisation affects egg traits, data on 718 great tit (Parus major) eggs from 90 clutches, spread across eight study sites in a city mosaic, were collected for two breeding seasons. All clutches were photographed, and analysed using digital imaging and visual scoring to assess egg volume and pigmentation patterns. Urbanisation was quantified as the percentage of Impervious Surface Area (ISA) in the vicinity of each clutch via satellite imagery.
3. In line with some of the earlier studies conducted on semi-natural bird communities, egg volume covaried with lay date and female body condition, while for both egg volume and egg pigmentation (spots percentage) a year effect was detected. However, in contrast with the predictions, there was no association between urbanisation and the examined egg traits.
4. While urban clutches are consistently smaller, this study shows that eggs as such are similar to those found in rural habitats in terms of volume and pigmentation patterns. Thus, results suggest that urban-driven environmental pressures may not be as strong or directional during the egg laying phase as they are at later stages of reproduction.

**Key words:** environmental constraints, calcium, egg pigmentation, egg volume, great tit, protoporphyrin IX, reproduction, urbanisation

**TABLE OF CONTENTS**

- **Text S1.** Descriptions of study sites set in a gradient of urbanisation in Warsaw, Poland.
- **Figure S1.** DHARMa diagnostics of models testing for the association between egg traits, urbanisation and life-history traits
- **Table S1.** Summary of ISA values for clutches from all study sites.
- **Table S2.** GLMMs with Gaussian distribution testing the association between egg pigmentation traits, urbanisation and life-history traits
- **Table S3.** Linear Mixed Effect Models (LMMs) testing the association between Impervious Surface Areas (%) measured in a 100m radius around each nestbox, and number of hatched offspring in great tits and blue tits. Based on data and methods from Szulkin & Corsini (2025).

**Text S1**

Descriptions of study sites set in a gradient of urbanisation in Warsaw, Poland. Descriptions are ordered from the most northern to the most southern sites. For more details see Corsini et al. (2020).

1. Suburban village (20°46'48.9748" E - 52°22'11.3382" N, *c*. 95 ha., 47 nestboxes). Palmiry village is located *c.* 21 km northwest from Warsaw city borders, next to Kampinos National Park. It is characterised by residential homes with gardens, crop fields and plots of unused land covered by grass, shrubs or trees.
2. Natural forest (20°47'14.3867" E - 52°21'22.5409" N, *c*. 38,500 ha., 110 nestboxes). Kampinos National Park is located *c.* 20 km northwest from Warsaw city border. The large mixed-coniferous forest, with a predominance of pine trees (*Pinus sp.*) and oaks (*Quercus sp.*), is divided into strictly and partially protected zones and is connected with Warsaw city by a green corridor.
3. Residential area I (20°57'39.37097" E - 52°16'23.71883" N, *c*. 19 ha., 52 nestboxes). Olszyna Residential Area is located in a north-western district of Warsaw. It is characterised by blocks of flats, public facilities (e.g., schools, grocery stores) and green spaces.
4. Urban woodland I (20°57'33.93652" E - 52°16'10.55093" N, *c.* 3.4 ha., 21 nestboxes). Olszyna woodland, adjacent to Residential area I, is a small urban wet forest with a predominance of common alders (*Alnus glutinosa*), birches (*Betula sp.*) and oaks (*Quercus sp.*).
5. Urban woodland II (20°58'23.44285" E - 52°14'52.45584" N, c. 33 ha., 91 nestboxes). Jewish Cemetery, located in the midtown of Warsaw, is a historical site, which was partially destroyed during World War II. A portion of the area has been in renovation since 1990s, however it mostly consists of moss-covered tombstones and a wild urban forest, composed of both native and foreign species, mainly oaks (*Quercus sp.*), birches (*Betula sp.*), elms (*Ulmus sp.*) and Norway maples (*Acer platanoides*).
6. Residential area II (20°59'5.74332" E - 52°14'52.17925" N, *c*. 36 ha., 46 nestboxes). Muranów Residential Area is located next to Urban woodland II. Similarly to Residential Area I, it is characterised by blocks of flats, public facilities and green spaces.
7. Urban park (21°0'6.98321" E - 52°12'46.66874" N, *c.* 65 ha., 105 nestboxes). Mokotów Field, located in the south-central district of Warsaw, is a large urban park characterised by a mosaic of flowerbeds, grass, trees as well as impervious surfaces. It also offers dining places, playgrounds, an outdoor gym and a water pond, making it a popular recreational area among city dwellers.
8. Office area (20°59'8.85224" E - 52°12'43.77676" N, c. 9 ha., 28 nestboxes). Ochota Campus, adjacent to the Urban park, is an office area consisting of university faculties, research centres, student dormitories and scarce greenery.

**Figure S1**. DHARMa diagnostics of models testing for the association between egg traits, urbanisation and life-history traits (see Methods).

(a)


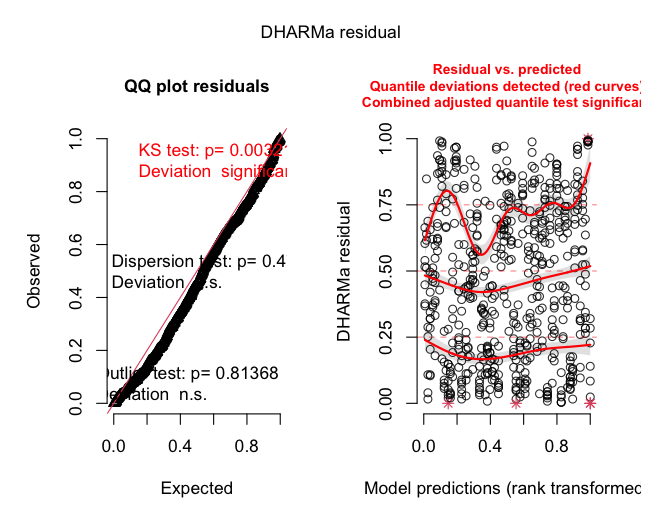


(b)


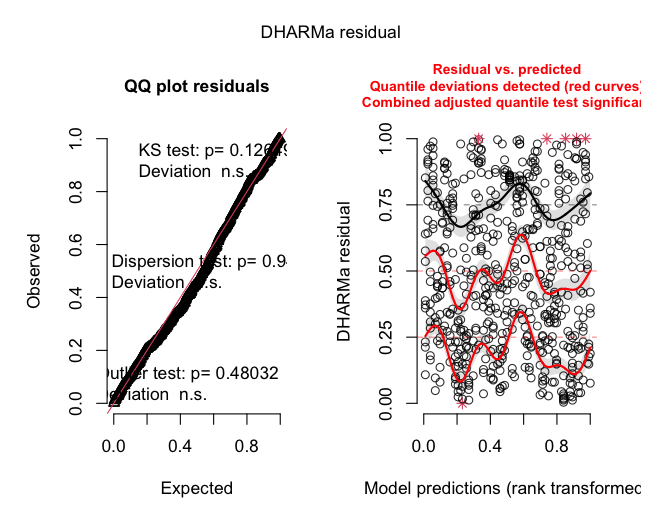


(c)


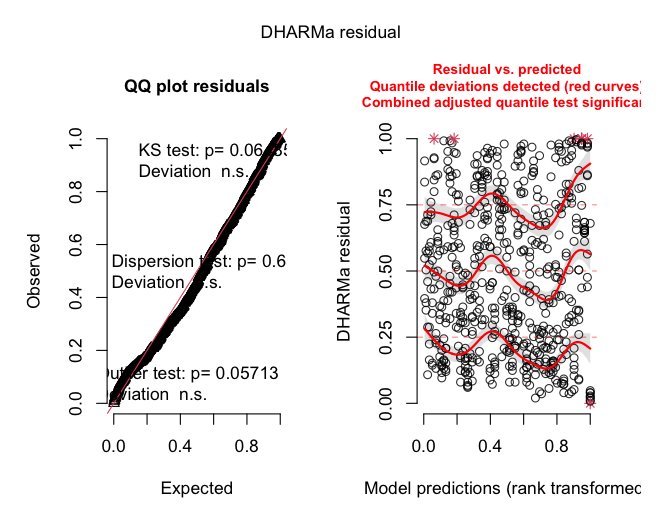


(d)


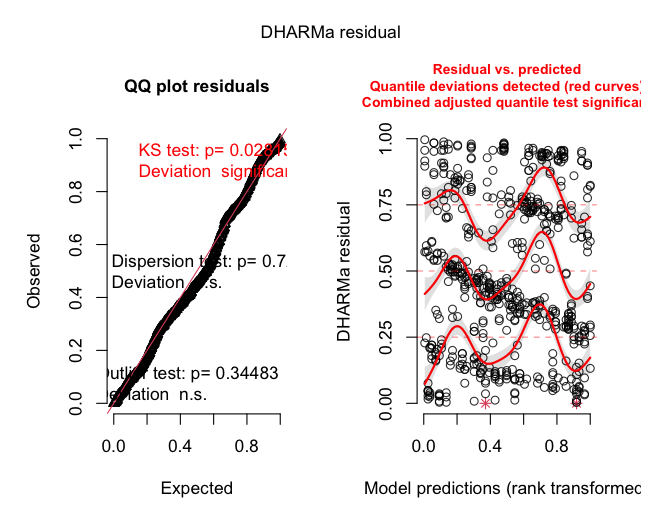


(e)


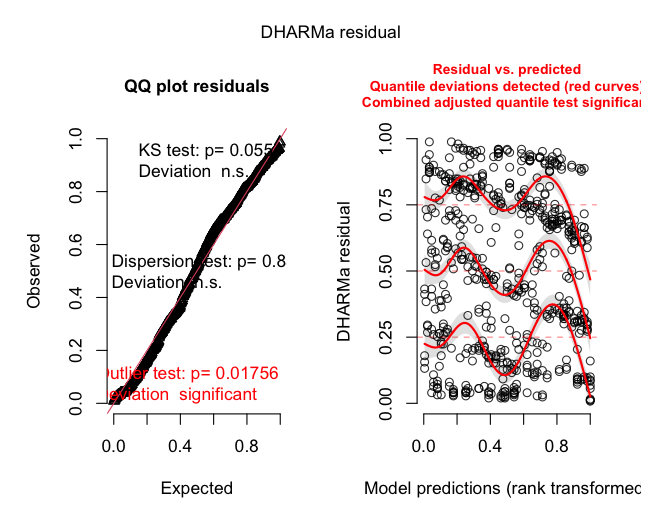


(f)


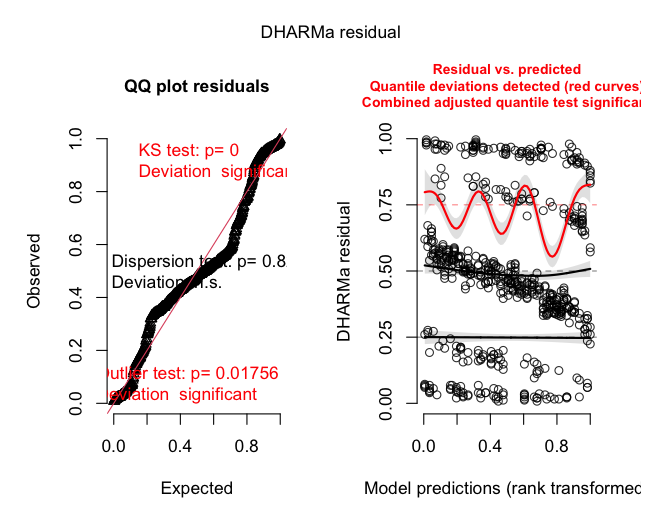


(a) *Model structure:* **Volume** ~ ISA_mc_ + Lay date_mc_ + Clutch size_mc_ + Female body condition_mc_ + Year, Random = Site ID (N=8) + Clutch ID (N=74) + Female ID (N=66)

(b) *Model structure:* **Spot red chroma** ~ ISA_mc_ + Lay date_mc_ + Clutch size_mc_ + Female body condition_mc_ + Year, Random = Site ID (N=8) + Clutch ID (N=74) + Female ID (N=66)

(c) *Model structure:* **Spot percentage** ~ ISA_mc_ + Lay date_mc_ + Clutch size_mc_ +Female body condition_mc_ + Year, Random= Site ID (N=8) + Clutch ID (N=74) + Female ID (N=66)

(d) *Model structure:* **Spot intensity** ~ ISA_mc_ + Lay date_mc_ + Clutch size_mc_ + Female body condition_mc_ + Year, Random = Site ID (N=8) + Clutch ID (N=74) + Female ID (N=66)

(e) *Model structure:* **Spot distribution** ~ ISA_mc_ + Lay date_mc_ + Clutch size_mc_ + Female body condition_mc_ + Year, Random = Site ID (N=8) + Clutch ID (N=74) + Female ID (N=66)

(f) *Model structure:* **Spot size** ~ ISA_mc_ + Lay date_mc_ + Clutch size_mc_ + Female body condition_mc_  + Year , Random = Site ID (N=8) + Clutch ID (N=74) + Female ID (N=66)

**Table S1**

Summary of ISA values for clutches for all study sites.

| Site | N_clutches_ | Minimum | Average | Median | Maximum |
| --- | --- | --- | --- | --- | --- |
| Suburban village | 18 | 0.00 | 1.97 | 0.85 | 13.60 |
| Natural forest | 16 | 0.00 | 0.00 | 0.00 | 0.00 |
| Residential area I | 2 | 32.38 | 42.06 | 42.06 | 51.74 |
| Urban woodland I | 2 | 24.19 | 24.19 | 24.19 | 24.19 |
| Urban woodland II | 3 | 10.24 | 18.99 | 16.65 | 30.07 |
| Residential area II | 5 | 35.62 | 42.91 | 43.68 | 47.17 |
| Urban park | 36 | 0.00 | 10.22 | 7.21 | 38.08 |
| Office area | 8 | 29.04 | 29.04 | 38.28 | 60.73 |
| Summary | 90 | 0.000 | 12.73 | 4.74 | 60.73 |

**Table S2**

GLMMs with Gaussian distribution testing the association between egg pigmentation traits, urbanisation and life-history traits, N_eggs_=568. Continuous predictors were mean-centered (mc). Significance levels are indicated **in bold:** ***p < 0.05, **p < 0.01, ***p < 0.001.**

| Family: Gaussian, Random = Site ID (N=8) + Clutch ID (N=74) + Female ID (N=66) | | | | |
| --- | --- | --- | --- | --- |
| Model structure (glmmTMB): Egg trait ~ ISA _mc_ + Lay date _mc_ + Clutch size _mc_ + Female body condition _mc_ + Year | | | | |
| **Spot red chroma** | Estimate | SE | z-value | p-value |
| Intercept | 0.41 | <0.01 | 164.53 | **<0.001***** |
| ISA | <0.001 | <0.001 | -0.37 | 0.714 |
| Lay date | <0.001 | <0.001 | 0.29 | 0.773 |
| Clutch size | <0.001 | <0.01 | -0.15 | 0.883 |
| Female body condition | <0.001 | <0.01 | -0.52 | 0.603 |
| Year | <0.001 | <0.01 | 0.31 | 0.754 |
| **Spot percentage** |  |  |  |  |
| Intercept | 17.65 | 0.92 | 19.29 | **<0.001***** |
| ISA | <0.01 | 0.03 | 0.14 | 0.887 |
| Lay date | 0.02 | 0.12 | 0.19 | 0.848 |
| Clutch size | 0.51 | 0.40 | 1.26 | 0.207 |
| Female body condition | -0.33 | 0.66 | -0.50 | 0.618 |
| Year | -7.66 | 1.18 | -6.51 | **<0.001***** |
| **Spot intensity** |  |  |  |  |
| Intercept | 3.26 | 0.14 | 23.45 | **<0.001***** |
| ISA | <0.01 | 0.01 | 0.63 | 0.526 |
| Lay date | -0.01 | 0.02 | -0.30 | 0.764 |
| Clutch size | 0.12 | 0.06 | 1.85 | 0.064 |
| Female body condition | -0.26 | 0.11 | -2.37 | **0.018*** |
| Year | -0.03 | 0.16 | -0.20 | 0.840 |
| **Spot distribution** |  |  |  |  |
| Intercept | 3.36 | 0.16 | 21.58 | **<0.001***** |
| ISA | -0.01 | 0.01 | -0.81 | 0.420 |
| Lay date | -0.02 | 0.02 | -0.73 | 0.463 |
| Clutch size | 0.10 | 0.07 | 1.45 | 0.146 |
| Female body condition | 0.15 | 0.12 | 1.26 | 0.207 |
| Year | -0.31 | 0.17 | -1.82 | 0.069 |
| **Spot size** |  |  |  |  |
| Intercept | 2.16 | 0.08 | 27.70 | **<0.001***** |
| ISA | <0.001 | <0.01 | 0.20 | 0.840 |
| Lay date | 0.01 | 0.01 | 0.91 | 0.362 |
| Clutch size | 0.05 | 0.04 | 1.32 | 0.186 |
| Female body condition | -0.01 | 0.06 | -0.21 | 0.84 |
| Year | -0.15 | 0.08 | -1.92 | 0.06 |

**Table S3.** Linear Mixed Effect Models (LMMs) testing the association between Impervious Surface Areas (%) measured in a 100m radius around each nestbox, and the number of hatched offspring in great tits and blue tits. Continuous predictors were scaled and mean-centered (mc). Significance levels are indicated **in bold:** ***p < 0.05, **p < 0.01, ***p < 0.001.** Data and details regarding methods can be found in Corsini & Szulkin (2025).

| Family: Gaussian, Random = Site (N=8) | | | | |
| --- | --- | --- | --- | --- |
| Model structure (LMMs): Number of hatched offspring ~ ISA _mc_ + Clutch size _mc_ + Female body mass _mc_ + Lay date _mc_ + Year | | | | |
|  | **Great tit (n = 252)** | | | |
| Variable | Estimate | se | t-value | p-value |
| (Intercept) | 7.78 | 0.20 | 38.28 | **<0.001***** |
| ISA | <0.01 | <0.01 | 0.13 | 0.899 |
| Clutch size | 0.95 | 0.05 | 17.87 | **<0.001***** |
| Female body mass | -0.07 | 0.09 | -0.80 | 0.426 |
| Lay date | -0.01 | 0.01 | -1.00 | 0.320 |
| Year _2017_ |  |  |  |  |
| Year _2018_ | 0.11 | 0.27 | 0.40 | 0.689 |
| Year _2019_ | -0.19 | 0.25 | -0.76 | 0.446 |
| Year _2020_ | -0.50 | 0.25 | -1.96 | 0.051 |
| Year _2021_ | -0.58 | 0.29 | -1.97 | 0.050 |
|  |  |  |  |  |
|  | **Blue tit (n = 305)** | | | |
| (Intercept) | 8.62 | 0.21 | 42.02 | **<0.001***** |
| ISA | -0.01 | 0.01 | -2.12 | 0.052 |
| Clutch size | 0.95 | 0.06 | 15.39 | **<0.001***** |
| Female body mass | -0.29 | 0.16 | -1.86 | 0.06 |
| Lay date | -0.01 | 0.02 | -0.56 | 0.57 |
| Year _2017_ |  |  |  |  |
| Year_2018_ | 0.40 | 0.28 | 1.43 | 0.154 |
| Year _2019_ | 0.25 | 0.28 | 0.89 | 0.373 |
| Year _2020_ | -0.34 | 0.28 | -1.22 | 0.222 |
| Year _2021_ | 0.18 | 0.29 | 0.63 | 0.503 |
